# Parkinson disease with constipation: clinical features and relevant factors

**DOI:** 10.1038/s41598-017-16790-8

**Published:** 2018-01-12

**Authors:** Qiu-Jin Yu, Shu-Yang Yu, Li-Jun Zuo, Teng-Hong Lian, Yang Hu, Rui-Dan Wang, Ying-Shan Piao, Peng Guo, Li Liu, Zhao Jin, Li-Xia Li, Piu Chan, Sheng-Di Chen, Xiao-Min Wang, Wei Zhang

**Affiliations:** 1 Department of Neurology, Beijing Tiantan Hospital, Capital Medical University, Beijing, 100050 China; 2 Department of Geriatrics, Beijing Tiantan Hospital, Capital Medical University, Beijing, 100050 China; 3 China National Clinical Research Center for Neurological Diseases, Beijing, 100050 China; 4 Key Laboratory for Neurodegenerative Disorders of the Ministry of Education, Capital Medical University, Beijing, 100069 China; 5 Center of Parkinson Disease, Beijing Institute for Brain Disorders, Beijing, 100069 China; 6 Beijing Key Laboratory on Parkinson Disease, Beijing, 100053 China; 7 Department of Neurobiology, Beijing Xuanwu Hospital, Capital Medical University, Beijing, 100053 China; 8 Department of Neurology, Ruijin Hospital Affiliated to Shanghai Jiaotong University School of Medicine, Shanghai, 200025 China; 9 Department of Physiology, Capital Medical University, Beijing, 100069 China

## Abstract

Constipation is one of the most frequent non-motor symptoms of Parkinson disease (PD) and it may be ignored by PD patients, leading to this problem not to be reported in time. The relationships between constipation and demographic variables, motor symptoms and other non-motor symptoms of PD are still unknown. PD patients were evaluated by diagnostic criteria of functional constipation in Rome III and divided into PD with constipation (PD-C) and PD with no constipation (PD-NC) groups. PD patients were assessed by rating scales of motor symptoms and other non-motor symptoms, activity of daily living and quality of life. The frequency of constipation in PD patients was 61.4%, and 24.5% of PD patients had constipation before the onset of motor symptoms. PD-C group had older age and age of onset, longer disease duration, more advanced disease stage, and more severe motor symptoms and non-motor symptoms, including worse cognition and emotion, poorer sleep quality, severer autonomic symptoms, fatigue and apathy. Binary Logistic regression analysis showed that the age, H-Y stage, depression, anxiety and autonomic dysfunction increased the risk of constipation in PD patients. Constipation exerted serious impact on the activity of daily living and quality of life in PD patients.

## Introduction

Parkinson disease (PD) is a common neurodegenerative disorder characterized by the progressive loss of neuromelanin-containing dopaminergic neurons in substantia nigra pars compacta (SNpc) and depletion of dopamine (DA) in striatum. PD was previously thought to be characterized by motor symptoms, including resting tremor, bradykinesia, rigidity, and gait and postural abnormalities. However, PD pathological stage based on the location of α-synuclein-immunopositive Lewy bodies^1^ indicates the presence of various non-motor symptoms, including autonomic dysfunction^2^, neuropsychiatric disturbance^3^, abnormal sensation^4^ and sleep disorders^5^, etc. Constipation is one of the most frequent non-motor symptoms in autonomic system^6,7^ and gastrointestinal disturbance of PD^8^. About 50^9^~80%^10^ PD patients suffer from constipation. It has been reported that constipation can precede motor symptoms by as much as 20 years^6^ and people with constipation may have a relatively high risk of developing PD^11^. Accordingly, constipation may predict the occurrence of PD. However, PD patients may not talk about their symptom of constipation actively, leading to this problem not to be reported in time^12^.

In previous studies, it was found that constipation was correlated with the duration and severity of PD^13^, and the frequency and severity of constipation were increased as PD progressed^14^. While constipation is one of the most studied prodromal symptoms of PD, how constipation is related to demographic variables, motor function and other non-motor symptoms of PD is still controversial so far.

In this study, we continuously recruited PD patients, evaluated clinical symptoms of PD and constipation by using a variety of related scales and analyzed the relationships among constipation, demographic variables, motor function, and non-motor symptoms in order to associate the clinical features and relevant factors of PD with constipation.

## Methods

### Ethics statement

The protocol was approved by the Institutional Review Board of Beijing Tiantan Hospital. All participants completed the written informed consents. This study met the guidelines of Capital Medical University, which abided by the Helsinki Declaration on ethical principles for medical research involving human subjects.

### Participants

Patients were diagnosed with PD according to UK Parkinson’s Disease Society Brain Bank criteria^15^. Total 306 PD patients were consecutively recruited from the Departments of Geriatrics and Neurology, Beijing Tiantan Hospital, Capital Medical University, from 2012 to 2016. PD patients with severe systemic diseases, such as anemia, heart failure, pulmonary disorders, chronic liver/renal failure, gastrointestinal disorders, infectious disease and chronic inflammatory disease were excluded, and patients with a positive family history of PD were also excluded.

Demographic variables, including gender, age, age of onset, disease duration, education level, side of onset, clinical type and levodopa equivalent daily dose (LEDD) were recorded for all participants.

Patients were evaluated by the professionally trained and qualified neurologists in our team and they did not know whether an individual patient had constipation or not.

### Constipation assessment

The Rome criterion is an internationally recognized objective definition of constipation and focuses on 6 symptoms, including straining, lumpy or hard stools, sensation of incomplete evacuation, sensation of anorectal obstruction or blockage, manual maneuvers to facilitate evacuation, and two or fewer bowel movements per week. It is associated with a diagnosis of constipation with 2 or more symptoms being present for at least 3 months^16^. According to the Rome criterion of constipation, patients in this study were divided into PD with constipation (PD-C) and PD with no constipation (PD-NC) groups. In PD-C group, we further asked about the frequency of daily bowel movement of each patient, additionally, the severity of constipation and the information on the use of laxatives were also collected.

### Assessments of clinical features

#### Motor function

The severity of PD was assessed by Hoehn-Yahr (H-Y) stage.

Motor symptoms of PD patients were evaluated by Unified Parkinson Disease Rating Scale (UPDRS) III, in which items 20 and 21 were for tremor, item 22 was for rigidity and items 23–26 were for bradykinesia. According to the method for clinical phenotypes classification by Schrag^17^, participants were divided into tremor type, bradykinesia-rigidity type and mixed type of PD.

Motor complications include wearing-off, on and off phenomenon and dyskinesia. Wearing-Off Scale was used for evaluating wearing-off phenomenon and UPDRS IV for assessing on-off phenomenon and dyskinesia.

#### Non-motor symptoms

Non-motor symptoms were screened by Non-Motor Symptoms Quest (NMSQ). It is a widely used^18^ self-administered 30-item instrument for screening the presence of a series of non-motor symptoms and calculating the incidence of each non-motor symptom.

Cognitive impairment was screened by Mini-Mental State Examination (MMSE) scale^19^, which is the most widely used by frontline physicians. MMSE covers the cognitive domains, including orientation, memory, attention, naming, repetition, comprehension, writing, and construction. The score ranges from 0 to 30 point(s). Patients with illiteracy, primary education, and above junior education are identified to have cognitive impairment when the score of MMSE scale is below 17, 20 and 24 points, respectively.

Hamilton Depression (HAMD) Scale -24 items is a frequently utilized depression severity rating scale^20^, which contains 24 variables, including depressed mood, guilt, suicide, initial insomnia, middle insomnia, delayed insomnia, work and interests, retardation, agitation, psychic anxiety, somatic anxiety, gastrointestinal symptoms, general somatic symptoms, genital symptoms, hypochondriasis, loss of weight, insight, diurnal variation, depersonalization, paranoid symptoms, obsessional symptoms, helplessness, hopelessness, and worthlessness. Score of HAMD Scale -24 items less than 8 points, between 8 to 19 points, 20 to 34 points and more than 35 points suggests no, mild, moderate and severe depression, respectively.

Hamilton Anxiety (HAMA) Scale -14 items is a clinician-rated scale and used to assess and quantify severity of anxiety^21^. The scale assesses a series of cognitive, behavioral, and somatic symptoms, including anxious mood, tension, fears, insomnia, cognitive impairement, depressed mood, somatic anxiety for muscle, somatic anxiety for sensation, cardiovascular symptoms, respiratory symptoms, gastrointestinal symptoms, genito-urinary symptoms, autonomic symptoms and behavior at interview. Clinicians are required to rate the severity of anxiety symptoms, which are scored on a 5-point scale ranging from 0 (absent) to 4 (severe). HAMD Scale -14 items less than 7 points, between 7–14 points and more than 15 points indicates no anxiety, possible anxiety and definite anxiety, respectively.

Pittsburgh Sleep Quality Index (PSQI) is a generic, self-applied questionnaire designed to evaluate mainly the quality of nocturnal sleep and to examine sleep habits and disturbances^22^ in the previous month. PSQI consists of 19 self-rated questions that are combined to form 7 component scores: subjective sleep quality, sleep latency, sleep duration, habitual sleep efficiency, sleep disturbances, use of sleeping medication and excessive daytime somnolence, each of which can be scored from 0 to 3 point(s) (from no difficulty to severe difficulty). Maximum score is 21 points, representing the maximum difficulty in 7 domains. Score of PSQI ≥5 indicates clinically meaningful sleep disorders.

Epworth Sleepiness Scale (ESS) is a self-administered scale, focusing on daytime sleepiness^23^. It consists of 8 items referred to daily or frequent situations, including sitting and reading, watching TV, sitting inactive in a public place (e.g. a theater or a meeting), as a passenger in a car for an hour without a break, lying down to rest in the afternoon when circumstance permit, sitting and talking to someone, sitting quietly after a lunch without alcohol and in a car, while stopped for a few minutes in the traffic. The response to each item rates the chance of dozing or sleeping in such situation. Item score ranges from 0 (would never doze or sleep) to 3 (high chance of dozing or sleeping) point (s) and the total scale score ranges from 0 to 24 point (s). ESS score >6 points represents no dozing or sleeping.

Scale for Outcomes in PD for Autonomic Symptoms (SCOPA-AUT) is a specific instrument designed to assess autonomic function for PD patients^24^. It is composed of 25 items, targeting the regions of gastrointestinal (7 items), urinary (6 items), cardiovascular (3 items); thermoregulatory (4 items), pupillomotor (1 item) and sexual (2 items for men and 2 items for women). Currently, there is no definite cut-off value for this scale. Generally, the higher the total score of SCOPA-AUT, the more severe the autonomic symptoms.

Fatigue is screened by Fatigue Severity Scale (FSS)^25^. It is a self-administered 9-item fatigue rating scale that encompasses several aspects of fatigue and their impact on the daily functioning of patients. Patients are asked to rate how each item describes their fatigue from 1 (“strongly disagree”) to 7 point (s) (“strongly agree”). Total FSS score is obtained by dividing the sum of all item scores by 9. Score of FSS ≥4 indicates clinically meaningful fatigue.

Restless Legs Syndrome (RLS) Rating Scale (RLSRS) is used to evaluate RLS for PD patients. RLSRS consists of 10 questions, needs face to face interview, and is rated from 0 to 4 point (s)^26^. RLSRS scores of 0, 1–10, 11–20, 21–30 and 31–40 points represents asymptomatic, mild, moderate, severe and very severe, respectively. It shows excellent clinimetric properties and has been validated in cross-sectional studies and applied in clinical trials.

Modified Apathy Evaluation Scale (MAES) is recommended to rate apathy in PD patients by Movement Disorders Society^27^. It is a well-validated and 14-item self-report tool, for example: are you interested in learning new things? Are you interested in anything? Do you care about your health? and so on. The score of MAES ranges  from 0 to 42 point (s), with higher score indicative of severer apathy^28^. Score of MAES ≥14 indicates clinically meaningful apathy.

#### Activity of daily living (ADL) and quality of life of PD patients

Activity of daily living is evaluated by ADL scale, which includes 20 items. The higher the score of the scale, the poorer the activity of daily living. Quality of life of PD patients was assessed by Parkinson Disease Quality of Life Questionnaire (PDQ) -39 items. The higher the score of the scale, the worse the quality of life. 

### Data analyses

Statistical analyses were performed with SPSS Statistics 20.0 (Chicago, IL, USA). P value was statistically significant when it was less than 0.05.

Demographic information, motor and non-motor symptoms were compared between PD-C and PD-NC groups.

Continuous variables, if normally distributed, were presented as means ± SDs and two groups were compared by 2-tailed t test. If not normally distributed, continuous variables were presented as median (quartile) and compared by nonparametric test. Discrete variables were compared by Chi square test.

Age, age of onset, disease duration, LEDD, H-Y stage, the scores of UPDRS III, UPDRS IV, MMSE, HAMD, HAMA, PSQI, SCOPA-AUT, FSS and MAES between PD-C and PD-NC groups were significantly different, thus, these variables were put into an established binary logistic regression equation and set as independent variables, whereas, with or without constipation in PD patients was set as a dependent variable. *P* value was significant when it was <0.05.

## Results

### The frequency and assessment of constipation in PD patients

In 306 PD patients, 188 cases (61.4%) were with constipation. Among 188 PD patients with constipation, 46 cases (24.5%) experienced constipation before the onset of motor symptoms. The evaluation of constipation in PD-C group was showed in Supplemental Table 1.

### Demographic information of PD-C and PD-NC groups

Demographic variables of PD-C and PD-NC groups were compared in Table 1. The age and age of onset of PD-C group were significantly older than that of PD-NC group (P < 0.01). The disease duration of PD-C group was dramatically longer than that of PD-NC group (P < 0.05). The LEDD of PD-C group was significantly higher than that of PD-NC group (*P* < 0.05). There were no significant differences in gender, educational level, side of onset and clinical type between PD-C and PD-NC groups (*P* > 0.05).

**Table 1:** Demographic variables of PD-C and PD-NC groups. **: *P* < 0.01; *: *P* < 0.05.

Variables	PD-C group (188 cases)	PD-NC group (118 cases)	P value
Male/total [cases/total (%)]	102/188 (54.3)	65/118 (55.1)	0.887
Age (years, mean ± SD)	64.71 ± 9.56	59.74 ± 10.71	0.000**
Age of onset (years, mean ± SD)	60.54 ± 10.32	56.52 ± 12.09	0.003**
Disease duration	3.00 (1.88~5.00)	2.00 (1.00~4.00)	0.006**
[years, median(quartile)]			
Education [cases/total (%)]			0.527
Primary school and below	54/188 (28.7)	32/118 (27.1)	
Middle and high school	98/188 (52.1)	57/118 (48.3)	
Bachelor’s degree and above	36/188 (19.2)	29/118 (24.6)	
Side of onset [cases/total (%)]			0.627
Left	84/188 (44.7)	51/118 (43.2)	
Right	100/188 (53.2)	66/118 (55.9)	
Both	4/188 (2.1)	1/118 (0.9)	
Clinical phenotype [cases/total (%)]			0.051
Tremor type	43/188 (2.9)	40/118 (33.9)	
Rigidity-bradykinesia type	22/188 (11.7)	17/118 (14.4)	
Mixed type	123/188 (65.4)	61/118 (51.7)	
LEDD (mg, mean ± SD)	276.36 ± 313.30	203.02 ± 276.60	0.033*

Abbreviations: LEDD = levodopa equivalent daily dose.

### Motor symptoms and motor complications of PD-C and PD-NC groups

Motor symptoms of PD-C and PD-NC groups were compared in Table 2. H-Y stage and UPDRS III score in PD-C group were remarkably increased when comparing with PD-NC group (*P* < 0.01), indicating that PD-C group had more advanced stage of PD and more severe motor symptoms.

**Table 2:** Motor symptoms and motor complications of PD-C and PD-NC groups. **: *P* < 0.01.

Variables	PD-C group (188 cases)	PD-NC group (118 cases)	P value
UPDRS III [points, median (quartile)]	30.3 (18.3~39.0)	23.6 (13.0~32.0)	0.000**
H-Y Stage [stage, median (quartile)]	2.0 (1.5~2.5)	1.7 (1.0~2.0)	0.000**
UPDRS IV [points, median (quartile)]	0.00 (0.00~2.00)	0.00 (0.00~1.00)	0.002**

Abbreviations: **UPDRS III** = Unified Parkinson’s Disease Rating Scale, Part III; **H-Y** = Hoehn and Yahr; **UPDRS IV** = Unified Parkinson’s Disease Rating Scale, Part IV.

Motor complications of PD-C and PD-NC groups were compared in Table 2. UPDRS IV score in PD-C group was significantly increased when comparing with PD-NC group (*P* < 0.01), implying that PD-C group had severer motor complications.

### Non-motor symptoms of PD-C and PD-NC groups

The numbers of non-motor symptoms of PD-C and PD-NC groups were compared in Supplemental Table 1. The total number of non-motor symptoms, the number of non-motor symptoms before and after motor symptoms in PD-C group were all significantly more than that of PD-NC group (*P* < 0.01).

The frequency of each non-motor symptom evaluated by NMSQ in PD-C and PD-NC groups was compared in Supplemental Table 2. The frequencies of dribbling, loss of taste/smell, swallowing/choking difficulties, constipation, bowel emptying incompletion, urine urgency, nocturia, apathy, hallucinations, sexual dysfunction, falls, insomnia, intense dreaming, rapid eye movement sleep behavior disorder, restless legs and diplopia, were all significantly increased in PD-C group compared with PD-NC group (*P* < 0.05).

The performance of each following non-motor symptom assessed by related rating scales in PD-C and PD-NC groups was compared in Table 3. Compared with PD-NC group, PD-C group scored conspicuously lower on MMSE scale and scored remarkably higher on the scales of HAMD, HAMA, PQSI, SCOPA-AUT, FSS and AS, suggesting that individuals in PD-C group had evidently worsened cognitive impairment, mood disturbances, sleep quality, autonomic symptoms, fatigue and apathy (*P* < 0.05).

**Table 3:** Non-motor symptoms of PD-C and PD-NC groups. **: *P* < 0.01.

Variables	PD-C group (188 cases)	PD-NC group (118 cases)	P value
MMSE score [points, median(quartile)]	27.00 (22.75~29.00)	28.00 (25.00~30.00)	0.009**
HAMD score [points, median(quartile)]	11.00 (5.00~19.00)	7.00 (3.00~12.00)	0.000**
HAMA score [points, median(quartile)]	9.00 (3.25~15.00)	5.00 (2.00~10.00)	0.000**
ESS score [points, median(quartile)]	5.00 (2.00~8.00)	4.00 (2.00~7.00)	0.228
PSQI score (points, mean ± SD)	8.11 ± 4.89	5.64 ± 3.84	0.000**
SCOPA-AUT score	42.00 (36.00~47.00)	37.00 (33.00~41.00)	0.000**
[points, median(quartile)]			
FS-14 score (points, mean ± SD)	9.01 ± 3.97	8.10 ± 4.26	0.061
FSS score [points, median(quartile)]	4.72 (3.00~6.11)	3.78 (2.19~5.03)	0.001**
RLSRS score [points, median(quartile)]	0.00 (0.00~14.00)	0.00 (0.00~10.00)	0.133
MAES score (points, mean ± SD)	17.97 ± 8.99	14.25 ± 8.91	0.001**

Abbreviations: **MMSE** = Mini-Mental State Examination; **HAMD** = Hamilton Depression Scale; **HAMA** = Hamilton Anxiety Scale; **ESS** = Epworth Sleepiness Scale; **PQSI** = Pittsburgh Sleep Quality Index; **SCOPA-AUT** = Scale for Outcomes in Parkinson’s Disease for Autonomic Symptoms; **FS** = Fatigue Scale; **FSS** = Fatigue Severity Scale; **RLSRS** = Restless Legs Syndrome Rating Scale; **MAES** = Modified Apathy Evaluation Scale.

Further comparison of detailed symptoms of autonomic dysfunctions by SCOPA-AUT scale in PD-C and PD-NC groups was performed in Supplemental Table 3. Compared with PD-NC group, PD-C group scored significantly higher on the gastrointestinal symptoms, urinary dysfunction, cardiovascular disturbance, and thermoregulatory dysfunction, demonstrating that PD-C group had severe gastrointestinal, urinary, cardiovascular, and thermoregulatory symptoms (*P* < 0.05).

There were no distinct differences in the scores of ESS and RLSRS between PD-C and PD-NC groups (*P* > 0.05).

### Activity of daily living and quality of life in PD-C and PD-NC groups

We conducted the comparisons of activity of daily living and quality of life in PD-C and PD-NC groups in Supplemental Table 4. Comparing with PD-NC group, the score of ADL scale in PD-C group was significantly increased (*P* < 0.01), demonstrating that PD-C group had obviously compromised activity of daily living.

The score of PDQ-39 score in PD-C group was markedly decreased compared with PD-NC group, implying that PD-C group had severely disturbed quality of life (*P* < 0.01).

### Risk factors of PD-C

Logistic regression analysis was performed to figure out the risk factors of PD-C in Table 4. Eventually, it was found that only SCOPA-AUT score was the risk factor of constipation in PD patients (OR = 1.091; *P* < 0.01).

**Table 4:** Risk factors of constipation in PD patients. *: *P* < 0.05.

Variable	B	OR value	95%CI	P value
Constant	−8.671			0.001
Age	0.070	1.073	0.871~1.321	0.509
Age of onset	−0.016	0.984	0.800~1.210	0.877
Disease duration	0.005	1.005	0.790~1.279	0.964
LEDD	0.000	1.000	0.999~1.002	0.826
H-Y Stage	0.528	1.695	0.846~3.395	0.136
UPDRS III score	0.000	1.000	0.962~1.041	0.981
UPDRS IV score	−0.038	0.962	0.753~1.230	0.760
MMSE score	0.025	1.025	0.928~1.133	0.625
HAMD score	−0.024	0.976	0.917~1.039	0.446
HAMA score	0.018	1.018	0.951~1.091	0.604
PSQI score	0.057	1.059	0.961~1.166	0.248
SCOPA-AUT score	0.088	1.091	1.031~1.155	0.003*
FSS score	0.024	1.024	0.846~1.241	0.805
MAES score	0.006	1.006	0.967~1.047	0.758

Abbreviations: LEDD = levodopa equivalent daily dose; **H-Y** = Hoehn and Yahr; **UPDRS III** = Unified Parkinson’s Disease Rating Scale, Part III; **UPDRS IV** = Unified Parkinson’s Disease Rating Scale, Part IV; **MMSE** = Mini-Mental State Examination; **HAMD** = Hamilton Depression Scale; **HAMA** = Hamilton Anxiety Scale; **PSQI** = Pittsburgh Sleep Quality Index; **SCOPA-AUT** = Scale for Outcomes in Parkinson’s Disease for Autonomic Symptoms; **FSS** = Fatigue Severity Scale; **MAES** = Modified Apathy Evaluation Scale.

## Discussion

In this study, 61.4% PD patients had constipation, in accordance with previous studies reporting 50% to 80% of constipation in PD patients^9,10^, thus, constipation is a common non-motor symptom of PD.

Here, it was found that 46 out of 188 patients (24.5%) in PD-C group experienced constipation before the onset of motor symptoms, illustrating that constipation is one of prodromal symptoms of PD. A previous study observed α-synuclein in colon tissue prior to onset of PD^29^, which might explicate constipation prior to motor symptoms.

Increasing evidence revealed that aging was an independent risk factor for the development and progression of PD^30^. In this study, it was observed that PD-C group had older age (Table 1). Additionally, PD-C group had older age of onset (Table 1), consistent with a study showing that constipation was more likely to occur in PD patients with disease onset at old-age^31^. Above data suggested that aging was involved in the constipation of PD. It could be speculated that in PD patients with older age and age of onset, less amount of activity and more weakened gastrointestinal motility evidently delayed colonic transport and thus caused constipation.

According to Braak stage of PD, Lewy bodies in the enteric nervous system and dorsal nucleus of vagus are associated with constipation of PD. In this study, PD-C group had longer disease duration (Table 1), more advanced disease stage and severer motor symptoms (Table 2). It was reported that constipation was positively correlated with disease duration and severity of PD^13^, and more severe with PD progression^14^. Thus, it might be that with PD duration and H-Y stage increased, dopamine level was markedly depleted and motor symptoms were remarkably aggravated as substantia nigra was largely impaired by Lewy bodies, which further precipitated gastrointestinal dysfunction and constipation deterioration.

Levodopa remains the mainstay of treatment for PD over 40 years after its introduction. Previous studies investigated the impact of dopaminergic treatment on autonomic symptoms, such as constipation, in PD patients^32,33^. In this study, PD-C group had significantly higher LEDD than PD-NC group (Table 1), this is in line with earlier studies reporting that changes in autonomic symptoms were related to dopaminergic treatment. In addition, the PD-C group had a longer disease duration and more advanced stages, thus might require more medications to alleviate symptoms.

Motor complications of PD are caused by the disease progression and long-time use of dopaminergic drugs with short half-life. In this study, PD-C group had more severe motor complications (Table 2), which might be explained by the findings that PD-C group had longer disease duration, more advanced H-Y stage, more severe motor symptoms, and larger dose of dopaminergic drugs.

In this study, PD-C group had more non-motor symptoms (Supplemental Table 5), among which constipation, ranked the top followed by taste/smell, dribbling, swallowing/choking difficulties, and nocturia, It was hypothesized that Lewy bodies, in addition to the deposition in the brain areas associated with constipation, were also extensively occurred in the brain regions associated with other non-motor symptoms.

Cognitive impairment was a very common non-motor symptom of PD^34^. In Braak stage V and VI, Lewy bodies occur in the brain regions related to cognitive impairment, such as limbic system and neocortex; meanwhile, Lewy bodies deposit more extensively in the areas associated with constipation, therefore, PD-C patients might suffered from significantly impaired cognitive function (Table 3).

Depression, a type of mood disturbance, might precede the development of motor symptoms and was considered as a prodromal symptom before the diagnosis of PD^35,36^. Anxiety is another common mood disturbance with high prevalence in PD patients despite that it was received attention in recent years^37^. In this study, depression and anxiety in PD-C group were significantly severer than that in PD-NC group (Table 3). It was reported that mood disturbances appeared to approximately double an individual’s risk of subsequent PD in a meta-analysis^36^. It is well known that 5-hydroxytryptamine (5-HT) is pivotal for the maintenance of normal mood, which depletion is one of the mechanism underlying depression and anxiety. Meanwhile, 5-HT exerted the action of enhancing gastrointestinal motility^38^, and 5-HT4 receptor agonists, mosapride^39^ and tegaserod^40^, alleviated the symptom of constipation. Accordingly, constipation and mood disturbances might share common mechanism relating 5-HT dysfunction in PD population.

Sleep disorders are the frequent non-motor symptoms in PD subjects^41^, and considered as an important independent determinant for impaired activity of daily living and quality of life^42^. In the current investigation, PSQI score in PD-C group was significantly higher than that in PD-NC group (Table 3), illustrating that the sleep quality was dramatically impaired in PD patients with constipation. Particularly, evaluations by using NMSQ showed that the incidences of insomnia, intense dreaming and rapid eye movement sleep behavior disorder in PD-C group were all significantly higher than that in PD-NC group (Supplemental Table 2), It was speculated that PD-C patients more easily suffered from above symptoms of sleep disorders, which might be due to discomfort in the abdomen caused by constipation.

PD patients with autonomic dysfunction presented numerous symptoms which compromised activities of daily living and quality of life^43,44^. The occurrence of autonomic symptoms is related to the deposition of Lewy bodies in the relevant brain regions. In this study, gastrointestinal, urinary, cardiovascular and thermoregulatory dysfunctions in PD-C group were significantly severer than that in PD-NC group (Supplemental Table 3) and high SCOPA-AUT score was the risk factor of constipation in PD patients (Table 4).

In gastrointestinal dysfunction, this study displayed remarkably higher incidence of dribbling, swallowing and bowel emptying incompletion in PD-C group than PD-NC group (Supplemental Table 2). Dribbling is not only caused by the increased secretion of salivary, but also by the reduced automatic swallowing due to dysphagia, resulting in a large amount of saliva fill in and outflow the mouth^45^. Dysphagia is related to the glossopharyngeum movement disorders, esophagectasis or slowdown of the esophageal peristalsis, and the decline of gastric motor function was frequently seen in advanced PD patients, which prolonged meal time and even leads to severe aspiration pneumonia and asphyxia^46^. Bowel emptying incomplete is diagnosed as constipation according to the Rome functional constipation diagnostic criterion, so the incidence of bowel emptying incompletion in PD-C group was significantly higher than that in PD-NC group.

The most striking feature of urinary dysfunction is the filling phase disorder, which includes urgency, nocturia and urinary incontinence^47^. Here, it was observed that the PD-C group had markedly higher incidences of urgency and nocturia than PD-NC group (Supplemental Table 2). Urgency and nocturia in PD patients might be attributed to detrusor hyperreflexia when the related brain region were affected by Lewy bodies.

The decreased cardiac uptake of ^123^I meta-iodobenzylguanidine (MIBG) on myocardial scintigraphy suggested that the degeneration of the cardiac sympathetic nerve began in the early stage of PD, even before neuronal loss in the dorsal vagal nucleus^48^. Data from this study implied that cardiovascular dysfunction in PD-C group was severer than that in PD-NC group (Supplemental Table 3), therefore, it was hypothesized that there was a common mechanism linking constipation and cardiovascular dysfunction in PD patients.

There was a paucity of literature about disruptions to thermoregulation in PD patients^49^. Firstly, PD patients are elderly population, which sensitivity to thermoreceptor is drastically decreased, accordingly, the process of heat production and heat radiation is drastically impaired when the outside temperature is changed. Secondly, sympathetic-adrenal system affected by Lewy bodies fails to control the process of heat production. Thirdly, severer motor symptoms, such as tremor and rigidity, cause skeletal muscle contraction and increase heat production. Above factors may contribute to the thermoregulatory dysfunction of PD patients. In this study, the results indicated that thermoregulatory dysfunction in PD-C group was severer than that in PD-NC group (Supplemental Table 3). Previous neuropathological studies found Lewy body deposition in the pons and medulla^50^, which might explain the thermoregulatory dysfunction and constipation observed in this study.

Fatigue was one of the most disabling non-motor symptoms^51^, however, how fatigue is related to constipation of PD is uncertain yet. Autonomic dysfunction was found to aggravate the subjective perception of fatigue^52^. The present study showed that PD-C group had severer fatigue than PD-NC group (Table 3), implying that constipation might aggravate fatigue of PD. When Lewy bodies deposited in raphe nuclei, locus coeruleus and magnocellular portions of the reticular formation, patients might manifest with autonomic dysfunction and fatigue, both of which might share similar pathological mechanism^53^.

Apathy, a lack of motivation characterized by diminished goal-oriented behaviors^54^, was one of the most common neuropsychiatric symptom of PD^55^. Here, apathy in PD-C group was dramatically severer compared with PD-NC group (Table 3), indicating that constipation might aggravate the symptom of apathy. A previous study found a negative correlation between apathy score and gray matter density in inferior frontal^56^ and premotor cortex, which were a part of autonomic system. Therefore, there might be a common structural basis for constipation and apathy for PD patients. Further investigation is needed to explore the underlying mechanism for their relationship.

Results from this study revealed that constipation dramatically decreased the activity of daily living and compromised the quality of life for PD patients. It was speculated that constipation together with more and severer motor and non-motor symptoms synergistically impact the activity of daily living and quality of life for PD patients.

In summary, this study systemically investigated the clinical characteristics and relevant factors of PD with constipation. The frequency of constipation in PD patients was 61.4%, and 24.5% of patients had constipation before the onset of motor symptoms. PD patients with constipation had older age and age of onset, longer disease duration, more advanced disease stage, and more severe motor and non-motor symptoms. Autonomic dysfunction increased the risk of constipation in PD patients. Constipation significantly compromised the activity of daily living and quality of life for PD patients.

## Electronic supplementary material

Supplementary Information
