# Supplementary material for: Parkinson disease with constipation: clinical features and relevant factors
Source: Sci Rep. 2018 Jan 12;8:567. doi: 10.1038/s41598-017-16790-8 (PMC5766597; doi:10.1038/s41598-017-16790-8)
Supplement: Supplementary file 1 — Supplementary Information [file 41598_2017_16790_MOESM1_ESM.doc]

**Parkinson disease with constipation:**

**clinical features and relevant factors**

Qiu-Jin YU^1^, Shu-Yang YU^2^, Li-Jun ZUO^1^,Teng-Hong LIAN^1^, Yang HU^2^, Rui-Dan WANG^1^, Ying-Shan PIAO^2^, Peng GUO^2^, Li LIU^2^, Zhao JIN^1^, Li-Xia LI^2^, Piu CHAN^7,5,6^ , Sheng-Di CHEN^8^, Xiao-Min WANG^9,4,5,6^, Wei ZHANG^2, 1, 3, 4, 5,6^

**Supplemental table 1 Evaluation of constipation for PD-C group**

|  |  | **cases** | percentage  (%) |
| --- | --- | --- | --- |
| Use of laxatives | yes | 88 | 46.8 |
|  | no | 100 | 53.2 |
| The severity of constipation  Frequency of bowel movements | straining  lumpy or hard stools  sensation of anorectal obstruction  with the aid of laxatives  once bowel movement/day  once bowel movement/2 days  once bowel movements/3 days  once bowel movements/＞3 days | 57  33  55  43  34  57  52  45 | 30.3  17.6  29.3  22.9  18.1  30.3  27.7  23.9 |

**Supplemental table 2 The frequency of non-motor symptom** of PD-C and PD-NC groups

| **NMS Quest**  [cases/total (%)] | **PD-C group**  **(188cases)** | **PD-NC group**  **(118cases)** | **P value** |
| --- | --- | --- | --- |
| Dribbling | 76/188 (40.4%) | 24/118 (20.3%) | **0.000**** |
| Loss of taste/smell  Swallowing/choking difficulties  Nausea/vomiting  Constipation  Fecal incontinence  Bowel emptying incomplete  Urine urgency  Nocturia  Pain  Weight loss/gain  Memory problems  Apathy  Hallucinations  Concentration problems  Depression  Anxiety  Sexual apathy  Sexual dysfunction  Orthostatic hypotension  Falls  Excessive daytime sleepiness  Insomnia  Intense dreaming  REM sleep behavior disorder  Restless legs  Leg swelling  Hyperhidrosis  Diplopia  Delusions | 86/188 (45.7%)  70/188 (37.2%)  44/188 (23.4%)  90/188 (47.9%)  8/188 (4.3%)  48/188 (25.5%)  51/188 (27.1%)  61/188 (32.4%)  43/188 (22.9%)  26/188 (13.8%)  76/188 (40.4%)  61/188 (32.4%)  15/188 (8.0%)  40/188 (21.3%)  62/188 (33.0%)  50/188 (26.6%)  34/188 (18.1%)  34/188 (18.1%)  31/188 (16.5%)  39/188 (20.7%)  51/188 (27.1%)  46/188 (24.5%)  54/188 (28.7%)  55/188 (29.3%)  47/188 (25.0%)  21/188 (11.2%)  45/188 (23.9%)  25/188 (13.3%)  8/188 (4.3%) | 33/118 (28.0%)  18/118 (15.3%)  21/118 (17.8%)  9/118 (7.6%)  1/118 (0.8%)  13/118 (11.0%)  20/118 (16.9%)  18/118 (15.3%)  17/118 (14.4%)  12/118 (10.2%)  35/118 (29.7%)  21/118 (17.8%)  2/118 (1.7%)  20/118 (16.9%)  34/118 (28.8%)  25/118 (21.2%)  12/118 (10.2%)  11/118 (9.3%)  16/118 (13.6%)  11/118 (9.3%)  26/118 (22.0%)  14/118 (11.9%)  19/118 (16.1%)  12/118 (10.2%)  16/118 (13.6%)  8/118 (6.8%)  25/118 (21.2%)  5/118 (4.2%)  1/118 (0.8%) | **0.002****  **0.000****  0.243  **0.000****  0.171  **0.002****  **0.040***  **0.001****  0.069  0.345  0.057  **0.005****  **0.020***  0.353  0.445  0.284  0.059  **0.035***  0.489  **0.009****  0.318  **0.007****  **0.012***  **0.000****  **0.016***  0.202  0.577  **0.009****  0.171 |

**Supplemental table 3 Autonomic Symptoms of PD-C and PD-NC groups**

| **Variable**  [cases/total (%)] | **PD-C group**  **(188cases)** | **PD-NC group**  **(118cases)** | **P value** |
| --- | --- | --- | --- |
| Gastrointestinal symptoms | 11.00 (9.00~12.00) | 8.00 (7.00~9.00) | **0.000**** |
| Urinary symptoms | 11.00 (8.00~14.00) | 9.00 (7.00~11.00) | **0.000**** |
| Cardiovascular symptoms  Thermoregulatory symptoms  Sexual symptoms | 4.00 (3.00~5.00)  6.00 (4.00~8.00)  4.00 (2.00~7.00) | 3.00 (3.00~4.00)  5.00 (4.00~7.00)  3.00 (2.00~6.25) | **0.016***  **0.032***  0.309 |

**Supplemental table 4 Activity of daily living and quality of life of PD-C and PD-NC groups**

| **Variable** | **PD-C group**  **(188cases)** | **PD-NC group**  **(118cases)** | **P value** |
| --- | --- | --- | --- |
| PDQL-39 (points, mean ± SD) | 127.98 ± 30.81 | 150.16 ± 27.42 | **0.000**** |
| ADL [points, median(quartile)] | 28.00（21.25~42.00） | 21.00（20.00~27.00） | **0.000**** |

Abbreviations: PDQ-39 = Parkinson’s Disease Quality of Life Questionnaire-39 items；ADL = Activity of daily living.

**Supplemental table 5 Numbers of non-motor symptoms of PD-C and PD-NC groups**

| **Variable** | **PD-C group**  **(188cases)** | **PD-NC group**  **(118cases)** | **P value** |
| --- | --- | --- | --- |
| Total numbers of NMS (cases, mean ± SD) | 11.1 ± 5.7 | 7.2 ± 4.8 | **0.000**** |
| Numbers of pre-MS NMS (cases, mean±SD) | 2.4 ± 2.6 | 1.3 ± 2.0 | **0.007**** |
| Numbers of post-MS NMS (cases, mean ± SD) | 8.7 ± 6.0 | 5.8 ± 4.5 | **0.002**** |

Abbreviations: **MS =** Motor Symptoms；**NMS** = Non-Motor Symptoms
